# Remote training for strengthening capacity in sexual and reproductive health and rights research: a systematic review

**DOI:** 10.1186/s12889-023-16851-w

**Published:** 2023-10-10

**Authors:** Carla Perrotta, Vicky Downey, Darin Elabbasy, Carolyn Ingram, Chungwan Lo, Amara Naseer, Anna Thorson, Vanessa Brizuela

**Affiliations:** 1 School of Public Health Physiotherapy and Sports Science, University College Dublin, ROI, Woodview House, Belfield, Dublin 4 Ireland; 2 UNDP/UNFPA/UNICEF/WHO/World Bank Special Programme of Research, Development and Research Training in Human Reproduction (HRP), Department of Sexual and Reproductive Health and Research, World Health Organization, Geneva, CH Switzerland

**Keywords:** Remote education, Sexual and reproductive health and rights, Research capacity strengthening

## Abstract

**Background:**

Training has been used to develop research skills among sexual and reproductive health and rights (SRHR) researchers. Remote education may accelerate transfer of skills and reduce barriers to strengthening research capacity. This systematic review aimed to assess the effectiveness of remote training on SRHR research and describe enablers and barriers of effective remote training.

**Methods:**

PubMed, Embase, and Scielo were searched up to December 2022 for studies that evaluated in any language online research training programmes either on a SRHR topic or tailored for professionals working in SRHR published since 1990. Characteristics of included studies, the programmes they evaluated, the programme’s effectiveness, and reported barriers and enablers to remote learning were extracted. Three researchers synthesized and described findings on effectiveness, impact and outcomes mapping them against the Kirkpatrick model. Additionally, thematic analysis from qualitative data was conducted to identify themes relating to the barriers and enablers of remote learning.

**Results:**

Of 1,510 articles retrieved, six studies that included 2,058 remote learners met the inclusion criteria. Five out of six studies described empirical improvements in participant research knowledge/skills and three studies reported improvements in attitudes/self-efficacy towards research. Follow-up surveys from four studies revealed frequent application of new research skills and improved opportunities for career advancement and publication following online trainings. Cited barriers to effective online SRHR research training included time management challenges and participants’ competing professional obligations; limited opportunities for interaction; and lack of support from home institutions. Cited enablers included well-structured and clear courses, learning objectives and expectations with participants; ensuring a manageable workload; facilitating interactions with mentors and hands-on experience; and selecting programme topics relevant to participants’ jobs.

**Conclusion:**

Remote SRHR training can lead to improvements in research knowledge, skills, and attitudes, particularly when course learning objectives, structure, and expectations are outlined clearly, and ongoing mentorship is provided.

**Supplementary Information:**

The online version contains supplementary material available at 10.1186/s12889-023-16851-w.

## Background

Strong research capacity is a key component to obtaining the evidence base for policies in pursuit of improved health outcomes [1]. One way on which research capacity has historically been strengthened has been through training, either via formal education degree programmes or short courses and workshops [2–4].

Strengthening capacity for sexual and reproductive health and rights (SRHR) research has the potential to contribute to decreasing existing inequities in the production of SRHR research as well as to supporting evidence based policy making and improved health outcomes [5]. SRHR research includes a variety of different topics that are inherently and politically charged in many environments, including abortion, family planning and contraception, gender and rights. SRHR research courses could cover commonly used methodologies in this field, including qualitative and quantitative methods or systematic reviewing specific to SRHR topics. These often-stigmatized topics pose additional challenges to learners [6–8].

Before the COVID-19 pandemic, most research capacity strengthening (RCS) learning activities in SRHR were conducted either face-to-face or through a blended format which combined remote learning and some in-person activities [9]. Emerging research suggests learning outcomes among healthcare professionals were achieved during the speedy shift towards online or other remote interactions during the COVID-19 pandemic [10–12]. Remote education is considered an approach that can potentially accelerate the transfer of skills and reduce some of the existing barriers in strengthening research capacity [3, 13]. The implementation of remote learning programmes can tame geographical barriers, increase student satisfaction, help reach a larger population, enhance collaborations, reduce costs, and give learners more control over their learning [14, 15].

Remote education fosters many of the principles of adult learning that posit that individuals learn by building on prior experiences, on their own belief systems, and on their autonomy and self-reliance, among others [16]. Remote learning platforms encourage learning by way of sharing opinions and ideas with others while simultaneously building connections with other online learners, enabling autonomous learning and allowing for the sharing of resources and experiences [17, 18].

Previous research has supported the use of remote learning to provide the opportunity to multiply access to education and facilitate contact with senior academics in other institutions [19–21]. Moreover, there is substantial evidence that healthcare professionals and to some extent undergraduate students participating in online education programmes can achieve similar learning outcomes as to face-to-face alternatives [20–22], and have strengthened sustainable research networks and communities of practice [23]. Remote education is not without challenges. For instance, educators require additional time to tailor material for different learners, there is a risk of information overload, as well as limited space for social interactions and networking. Furthermore low internet connectivity remains in many world regions and healthcare professionals may not be granted protected time for training [24–27].

Irrespective of the reported benefits and barriers of online learning, the effectiveness, potential impact and sustainability of fully remote training programmes in SRHR remains unclear [28], particularly given inherent sensitivities that surround many SRHR related issues in certain contexts, including sexuality and sexual health, abortion, contraception, and violence, among others [29]. Despite the rise in both local and global initiatives to expand RCS activities through training and education, there still remains a level of uncertainty on how to best strengthen and deliver capacity strengthening learning activities [30], and on how to ensure the goals achieved are sustained and result in improved outcomes [31].

This study aimed to conduct a systematic review to describe the effectiveness of remote education programmes to strength research capacity in SRHR, as well as to describe enablers and barriers linked to remote training from the perspective of researchers and study participants. We consider remote education as any training (exclusive of degree programmes) that is offered using the internet or other remote connectivity options, whether synchronous or asynchronous either on SRHR specific topics or research training tailored to SRHR professionals. The review contributes to the body of literature on SRHR RCS and sheds light on remote education as a potential strategy to overcome the challenges associated with sustainable training efforts, particularly among researchers in low- and middle-income countries.

## Methods

We conducted and reported the systematic review following the Preferred Reporting Items for Systematic Reviews and Meta-Analyses (PRISMA) (See Additional File 1). The review protocol was registered in International Prospective Register of Systematic Reviews database (PROSPERO, CRD42022328417). Additionally, in accordance with SAGER guidelines for reporting sex and gender information in studies, this review was designed to include studies without discrimination based on sex or gender and to analyse gender participation and differences in effectiveness by gender in the included studies [32].

### Search strategy and screening

We searched the literature for articles and conference proceedings published in any language in three electronic databases (PubMed, Embase, and Scielo), from January 1990 to December 2022. The strategy was developed with the assistance of a university librarian. The search terms were performed individually and then combined across the electronic databases. We searched for studies focusing on research methods training on a wide range of sexual and reproductive health and rights (Table 1). Studies yielded by the search strategy were downloaded and imported into Covidence (Veritas Health Innovation, Melbourne, Australia) and independently screened by two reviewers in accordance with the eligibility criteria. Three researchers (VD, CI, and DE) screened imported studies based on title and abstract and full text review. Two reviewers (VB, CP) addressed disagreements. Suitable studies were included for data extraction and reasons for exclusion were documented. The search was supplemented with forward and backward chain search in references lists from the identified eligible articles using the “Connected Papers” website (https://www.connectedpapers.com/).

**Table 1:** Search strategy

(“Health Care practitioners”) OR (“health personnel”[MeSH Terms]) OR (researcher)
AND
(reproductive health) OR (sexual health) OR (reproductive health right*) OR (sexual right*) OR (reproductive right*) OR (sexual function) OR (sexual satisfaction) OR (sexuality) OR (gender-based violence) OR (violence against women) OR (gender-based coercion) OR (sexual coercion) OR (contracept*) OR (antenatal) OR (childbirth) OR (postnatal) OR (maternal health) OR (perinatal health) OR (abortion) OR (family planning) OR (adolescent pregnancy) OR (teenage pregnancy) OR (sexually transmitted infection*) OR (sexually transmitted disease*) OR (bacterial vaginosis) OR (chlamydia) OR (gonorrhoea) OR (hepatitis) OR (herpes) OR (HIV) OR (AIDS) OR (human papillomavirus) OR (HPV) OR (pelvic inflammatory disease) OR (infertility) OR (syphilis) OR (trichomoniasis) OR (reproductive cancer*) OR (cervical cancer) OR (ovarian cancer) OR (uterine cancer) OR (vaginal cancer) OR (vulvar cancer) OR (fallopian tube cancer) OR (endometriosis) OR (female genital mutilation) OR (FGM) OR (gender equality) OR (lesbian) OR (gay) OR (bisexual) OR (transgender) OR (queer) OR (premature birth) OR (neonatal mortality) OR (stillbirths) OR (partograph*) OR (Gender) OR (Sex)
AND
((research capacity building) OR (capacity building) OR (research capacity) OR (research training) OR (research education) OR (research learning) OR (research capabilities) OR (systematic review training) OR (systematic review learning) OR (scoping review training) OR (scoping review learning) OR (meta-analysis training) OR (meta-analysis learning) OR (qualitative methods) OR (quantitative methods) OR (qualitative research training) OR (quantitative research training) OR (research leadership) OR (research methods) OR (research implementation) OR (Research) OR (research methods) OR (epidemiology) OR (qualitative research)) OR (program*) OR (Training) OR (research)
AND
(“remote learning”) OR (“remote education”) OR (“remote teaching”) OR (“remote course*”) OR (“remote lectur*”) OR (“remote training”) OR (“remote instruction*”) OR (“distance education”) OR (“distance learning”) OR (“distance teaching”) OR (“distance course*”) OR (“distance lectur*”) OR (“distance training”) OR (“web-based learning”) OR (“web-based education”) OR (“web-based teaching”) OR (“web-based course*”) OR (“web-based lectur*”) OR (“web-based training”) OR (“web-based instruction*”) OR (“online learning”) OR (“online education”) OR (“online teaching”) OR (“online course*”) OR (“online lectur*”) OR (“online training”) OR (“online instruction*”) OR (“blended learning”) OR (“blended education”) OR (“blended teaching”) OR (“blended course*”) OR (“blended training”) OR (“blended instruction*”) OR (“computer assisted instruction”) OR (“computer assisted learning”) OR (“computer assisted teaching”) OR (“computer assisted education”) OR (“online workshop*”) OR (“web-based workshop*”) OR (“education, distance”[MeSH Terms]) OR (“Massive online courses”) OR (e-learning) OR (elearning) OR (“Computer-Assisted Instruction/methods”[MAJR])

### Eligibility criteria

Studies that met the following criteria were eligible for inclusion in the review:

- *Type of participants:*
- Adults (18 years of age or older) participating in an online/remote training/education programme on sexual and reproductive health and rights research methods.
- *Type of studies:*
- We sought to include (1) Randomized controlled trials, (2) Quasi-randomised, (3) Cluster randomized, and (4) non-randomized studies: non-randomized controlled trials, interrupted time series, controlled before-and-after studies, cohort studies, mixed-method studies, or cross-sectional/cross-sectional descriptive studies.
- *Exclusion criteria:*
- Studies that met the following criteria were excluded: (1) protocols of future studies, (2) studies that were not targeted to SRHR research education, (3) studies where the training was designed to gain a clinical skill, (4) descriptions of remote training experiences without a report on at least one measure of effectiveness, (5) studies focusing on degree programmes (e.g., online master’s or doctoral degrees).

### Data extraction

A structured data extraction form was developed and piloted by the reviewers. Three reviewers (VD, DE, and CI) extracted data from eligible studies and a third reviewer (CP) assessed discrepancies. Data pertaining to study design and characteristics of educational activities were retrieved. Any discrepancies throughout the data extraction process were resolved through discussion among the research team until consensus was reached.

We gathered data on the academic institution hosting the training activity, country of origin of attendees as well as the expected learning outcomes and topic covered. We also retrieved information on how the course or module was developed and by whom, how the education programme was implemented (e.g., blended, fully online, a-synchronic or synchronic sessions), availability of mentorship, how the remote education platform and course were implemented, and duration.

We adapted concepts from the Kirkpatrick model to evaluate outcomes of education and training programmes which includes four levels for assessing these results: reaction, learning, behaviours, and results [33]. For this analysis, the following effectiveness measures were extracted if available: learner satisfaction; assessment of knowledge gained, self-reported knowledge, skills gained; belief about capabilities and intentions to apply knowledge; and lastly, impact measures (e.g., career advancement, grant applications, number of publications).

Subsequently, we searched for either reported enablers of remote learning as well as barriers and challenges. We used the definition study authors provided for enablers, which included factors that were either reported directly from the participants through open-ended exit surveys, interviews or focus groups, or as reflected upon by the study authors or programme implementers as being beneficial to achieve the desirable learning outcomes. Barriers and challenges were defined as obstacles that prevented learners from reaching the intended educational goals.

### Data collation and analysis

The initial intention of this systematic review was to conduct a meta-analysis of effectiveness measures. However, given the limited data, three researchers (VD, DE, and CI) synthesized and described the findings on effectiveness and impact.

In order to identify themes relating to enablers and challenges of online education (through exit surveys, students interviews, authors’ reflections) two researchers (VD and CI) followed the traditional steps of thematic analysis: getting familiar with the data -reporting or discussion-, initial coding, searching for themes, reviewing themes and charting and compiling the data aligned with our study objectives [34].

### Quality assessment

Quality assessment was undertaken to evaluate the methodological quality of the studies by two reviewers (AN, CI). The NIH quality assessment tool was used for before and after and cross-sectional studies [35].

## Results

### Study characteristics

**Fig. 1:**
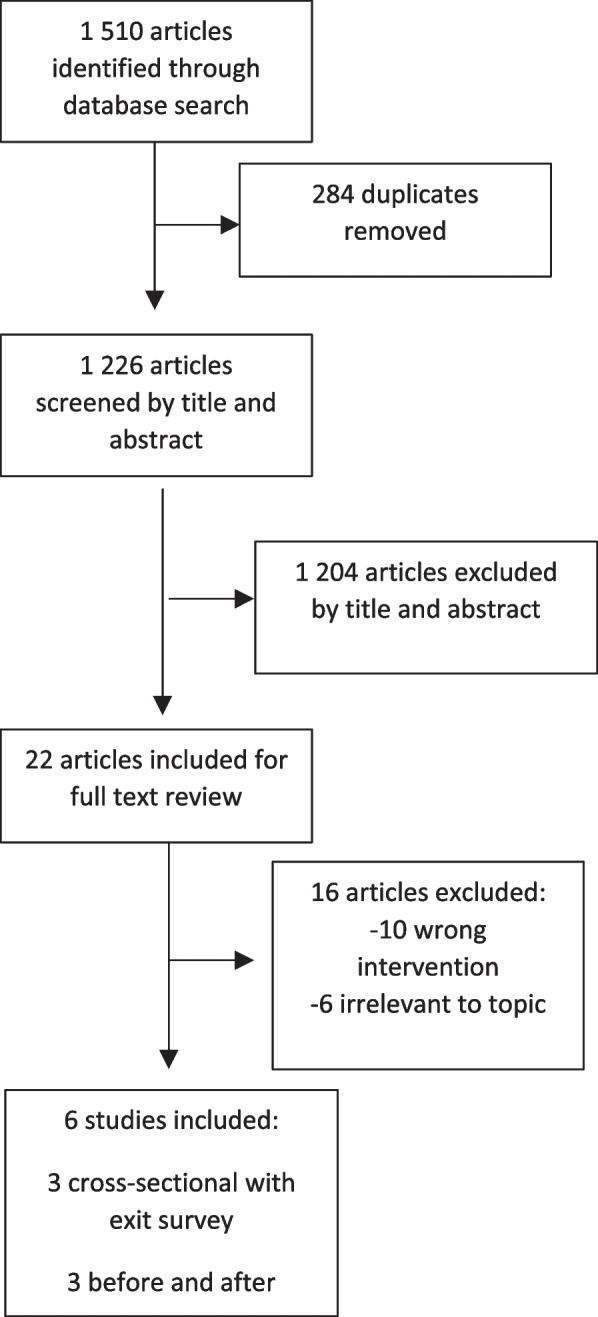
Flow chart of included papers

Of 1,510 identified studies, six met the inclusion criteria [36–41] (Fig. 1). Two were published as abstract proceedings [36, 37]. The SRHR dimension of selected studies was either a programme tailored to professionals working on reproductive health or the host institution remit focused on sexual and reproductive health or gender studies.

Study designs included three studies with before and after surveys [39–41] and three cross-sectional exit surveys [36–38] (Table 2). Three out of six studies were ranked as ‘Good Quality’ while the remaining studies were ranked as ‘Fair Quality’ (Table 2). In all remote programmes, the hosting institutions belonged to a high-income country (four US, one Canada) or an international organization based in Geneva, Switzerland. Two remote learning initiatives included only learners from the USA, while others collected data from participants from multiple countries.

**Table 2:** Study characteristics

**Author**	**Organizing Institution**	**Study Design**	**Participants (type and countries); course completion rate**	**Programme topic**	**Type of Programme and duration; Iterations**	**Outcome**	**Quality Assessment**
Abawi 2016 [38]	Geneva Foundation for Medical Education and Research (GFMER)	Cross-Sectional	Nurses (*N* = 13), Midwives (*N* = 4), Medical Doctors (*N* = 106), Public Health (*N* = 17) and other Health Professionals (*N* = 35) from 45 countries globally (Total *N* = 175); 65% (219/337)	Research methods in SRHR	Online/W b-based Course 8 months; two	Gained knowledge and skills through self-reported survey	Good^a^
Argawal 2021 [39]	American Center for Reproductive Medicine	Before & After	Medical Students & Medical Doctors from USA, India, Iran, Algeria (*N* = 28); 100% (28/28)	Mentorship programme structured around scientific writing projects	Online/Web-based Course 6 weeks; one	Increased knowledge and satisfaction through self-reported survey	Fair^a^
Brickley, 2018 [36]	University of California, San Francisco (UCSF) International Traineeships in AIDS Prevention Studies (ITAPS)	Cross-Sectional	Scientists from low and middle income countries (*N* = 37); 100% (37/37)	Training in research methods and scientific writing	Blended Course/Workshop 1 Year; three	Increased capacity to develop tables/figures and writing sections of a scientific manuscript	Fair^a^
Farel, 2001 [40]	Department of Maternal and Child Health (MCH), University of North Carolina at Chapel Hill	Before & After	MCH Public Health Professionals from USA (*N* = 28); 70% (28/40)	Analytic skills training	Online/Web-based Course 1 Year; one	Improved knowledge, beliefs, self-efficacy, skill level, and practice of the skills taught as measured through pre-test and post-tests	Fair^a^
Santoro 2021 [37]	Duke University Clinical Research Training Program [41]	Cross-Sectional	Reproductive Endocrinologist Subspecialists (including urologists and other Ob-Gyn subspecialists) from USA (*N* = 22); not reported	Clinical research training	Online/Web-based Course 2 Years; thirteen	Reported course satisfaction through an online survey	Good^a^
Tannenbaum & van Hoof, 2018 [41]	Institute of Gender and Health, Canadian Institutes of Health Research,	Before & After	Total numbers of learners was 1441, Researchers (*N* = 808), Researchers and Peer Reviewers (*N* = 256), Trainees (*N* = 158), Government Employees (*N* = 122) and other Health Professions (*N* = 140) form Canada, USA and other countries in Asia and Europe	Methods for integrating and appraising sex and gender variables in health research	Online/Web-based Course 1 year	Improved knowledge and self-efficacy	Good^a^

^a^ Score calculated using NIH quality assessment tool (See Additional File 2)S)

The total sample of learners was 2,058. Of the included six studies, only two studies reported the gender of participants. In one study the majority of participants were males 56.1% (*N* = 113/175) [38], while the other study reported a higher proportion of females 78.6% (*N* = 22/28) [39].

Targeted learners were researchers, government officials, public health officials and healthcare professionals. Participants in the reported studies were researchers (*N* = 1164), government employees (*N* = 61), healthcare professionals (*N* = 173), maternal and child health (MCH) epidemiologists (*N* = 347), scientists (*N* = 37), and medical students (*N* = 28) (Table 2). The type of training provided was primarily continuing education and aimed to improve research methodology tailored for either SRHR topics or professionals working on SRHR [36–38], scientific writing [36, 39], methods for integrating sex and gender variables in health research [41] and data analysis skills for professionals working in SRHR [40].

Course duration varied, ranging from 6 weeks up to two-years long. Programme completion rates, where reported, ranged from 65% (219/337) [38], 70% (28/40) [40] to 100% (28/28, 37/37) [36, 39]. The earliest included course began in 2001 [40], whereas some of the online programmes are still ongoing [39, 41].

### Development and implementation of the programme

The methods used by various organizations and institutions to develop, implement, and evaluate online research methods training are described in Table 3. All six included programmes targeted health professionals, researchers involved in sexual and reproductive health work who had demonstrated interests or needs in enhanced research skills in SRHR. The curricula for the six programmes were designed by academic experts working for, or in partnership with, the organization in which each respective programme was based (e.g., the Duke University Clinical Research Training Program). Online lectures were provided in real-time [39], or pre-recorded to accommodate different time zones and schedules [38, 41]. Two programmes included a face-to-face component; this involved a 3-week writing sabbatical at the University of California, San Francisco [36], and in-person meetings with researchers conducting clinical trials in the United States [37]. Three initiatives specified assigning scholars with mentors [36, 38, 39]; one study mentioned the provision of detailed feedback to scholars from programme faculty [40]. Of note, none of the included studies described massive open online courses (MOOC).

**Table 3:** Description of implemented sexual and reproductive health and rights online research training programmes

**Author**	**Intended Participants**	**Recruitment methods**	**Programme Design**	**Programme Format**	**Programme Content**	**Additional supports**	**Methodology to assess effectiveness & impact**
Abawi 2016 [38]	Health professionals involved in sexual and reproductive health work Focus on developing countries	Geneva Foundation for Medical Education and Research (GFMER) website Promotion by country coordinators, GFMER members and partner institutions	Modules designed by experts in the field based on WHO SRH guidelines	Pre-recorded lectures	Five core modules on sexual and reproductive health and rights topics Two additional modules on research methodology and sexual and reproductive rights	Reading materials Referrals to websites Online community using Google Groups, social media Personal coach assigned to each learner	Online questionnaire distributed two years after the programme with questions on: 1-Use of knowledge acquired 2-Teaching and publication opportunities
Agarwal 2021 [39]	Medical trainees, physicians and residents with an interest in developing research skills	Online applications (dissemination channels not specified)	International team of clinicians and scientists with expertise in clinical practice, scientific publication and teaching programmes under the guidance of American Center for Reproductive Medicine (ACRM) management	Weekly live lectures and discussion sessions Weekly writing tasks Development of a final writing project	Scientific Writing, Scientific Methodology Understanding, Soft Skills Development, Andrology Knowledge, and Plagiarism Understanding 6-week writing project	One-student – one mentor strategy Social interactive programmes following lectures Recognition for top students	Weekly surveys assessing teaching and learning process Exit survey with pre- and post-self-reported analysis of learning outcomes (0–5 Likert Scale)
Brickley2018 [36]	Scientists from LMICs	International AIDS Vaccine Initiative (IAVI) Network	Structured curriculum developed by ITAPS and mentors-in-training	Structured coursework (details unspecified) 3-week writing sabbatical at UCSF	Scientific writing	One mentor for every 2–3 trainees	Post-course kills assessment conducted by mentors Focus groups Surveys Number of Publications
Farel 2001 [40]	State and local health department staff members	Via state and local health departments	Faculty members with expertise in specific content areas	Six, 1-month modules	Basic epidemiological and statistical concepts Qualitative data collection and analysis Measurement of social inequalities in health Development of data collection instruments and planning data analysis Economic analysis Use of GIS software	Feedback from faculty	Pretest/Posttests conducted online immediately before and after completion of each module to measure knowledge, beliefs, self-efficacy, and general skills 6-month follow-up survey on frequency of practice, application of skills
Santoro 2021 [37]	Reproductive Endocrinologists, urologists, and Ob-Gyn specialists seeking to develop clinical research skills	Online applications (dissemination channels not specified)	CREST faculty and staff	First year: Didactic online training Year two: Research mentorship	Clinical research methods	Biostatistical support, opportunities to engage in team science CREST seminars In-person investigator meetings with NIH PIs	Online survey on satisfaction with the programme and levels of support Scholar interviews centered on experiences engaging with the programme, career advancement, and research skills gained
Tannenbaum & van Hoof, 2018 [41]	Public health researchers and government employees	Emails, newsletters, social media platforms subscribers, presentations, flyers, and as a mandatory requirement for funding provided by the institute of Gender and Health	Consultation with members of the Advisory Board of the Institute of Gender and Health and experts	A set of interactive Internet-based e-learning courses with pre and post tests	Three modules on Biomedical research, Data collection in humans and analysis of human data, designed to close knowledge, skill, and attitude gaps relating to sex and gender concerns in health research	No additional support was offered	Pre- and post-tests were conducted to online to test knowledge, skills, and self-efficacy Access to the post-test requires completion of one of the online learning modules

### Main findings on effectiveness and impact

Diverse methods were used to evaluate each programme’s effectiveness and impact. One study distributed an exit survey with retrospective pre-test and post-test self-evaluations of learning outcomes using 5-point Likert scales (*N* = 28) [40]. Another distributed pre-test/post-test self-assessments immediately before and after the completion of each module to measure knowledge (5-point Likert scale, *N* = 28), beliefs and self-efficacy (4-point Likert scale, *N* = 28), and skill level (7-point Likert scale, *N* = 23) [39]. Four studies conducted follow-up surveys to measure participant satisfaction, career advancement, and application of research skills (*N* = 257) [38–41]; two ran focus groups or interviews (*N* = 59) [36, 37], and two measured impact in terms of number of publications from participants (*N* = 384) [36, 38] (Table 3).

#### Increased satisfaction (level 1)

Only one of the identified studies reported participant satisfaction. Sixty-four percent (64.3%, *N* = 18/28) of learners strongly agreed and 35.7% (*N* = 10/28) agreed with regards to satisfaction in achieving their expected learning outcomes [39] (Table 4).

**Table 4:** Summary of reported effectiveness and impact of remote research training in six included studies^a^

***Theme***	***Description***	***Contributing papers***
*Reaction: Satisfaction*		
High satisfaction	Course learners were asked through a survey to agree, strongly agree, disagree or have a neutral stand on statements related to satisfaction and course content. The majority of learners have expressed satisfaction regarding achieving the expected learning outcomes of the course (64.3% strongly agree and 35.7% agree)	Agarwal 2021 [39]
*Learning: Knowledge and skills*		
Improvements in knowledge	Pretest/posttest assessments show that participants’ knowledge on basic epidemiological and statistical concepts, qualitative and quantitative data collection and analysis, measurement of social inequalities in health, and/or SRH topics improved significantly from the programme	Agarwal 2021 [39] Farel 2001 [40]; Tannenbaum & van Hoof, 2018 [41]
Improvements in skills	Pretest/posttest assessments, follow-up surveys, and scholar interviews show that participants’ skills in scientific writing, scientific methodology understanding, and/or data analysis and interpretation improved significantly from the programme (i.e., average self-reported skill levels on a 5- or 7-point Likert scale, high percentage of participants report that skills were acquired)	Agarwal 2021 [39] Farel 2001 [40]; Tannenbaum & van Hoof, 2018 [41]
Improvements in self-efficacy and attitudes towards remote learning	Pretest/posttest assessments and follow-up surveys show that participants’ self-reported self-efficacy (i.e., belief in their own competence to perform a particular research task), interest in and motivation to apply acquired knowledge and skills to their research improved significantly from the programme	Farel 2001 [40]; Santoro 2021 [37]; Tannenbaum & van Hoof, 2018 [41]
Application of skills	Most participants had the opportunity to teach (39%, *N* = 69/174 and/or share (74%, *N* = 17/22 newly acquired research skills with colleagues, or to apply them to their own research projects (98%, *N* = 171/174; 82%, *N* = 18/22 following completion of the programme	Abawi 2016 [38]; Farel et al., 2001 [40] Santoro 2021 [37]
Research career advancement	Many participants felt the course had helped advance their career (81%, *N* = 142/174 and/or had a scientific paper published or in peer review (47%, *N* = 82/174); 74%, *N* = 27 as a result of having completed the programme	Abawi 2016 [38]; Agarwal 2021 arg [39]; Brickley 2018 [36]; Santoro 2021 [37]

^a^ Organization of themes and topics based on Kirkpatrick’s four levels model [33]

#### Improvements in knowledge and skills (level 2)

Five out six studies reported empirical improvements in participant research knowledge and/or skills [37–41] (Table 4). One programme [38] found that knowledge on research methods across six month-long modules improved by 0.75 out of 5 points on average (SD =  ± 0.90), a difference that paired samples *t* tests revealed to be statistically significant (*N* = 28, *p* < 0.0001) indicating knowledge gain across research methods topics (i.e., basic epidemiological and statistical concepts, qualitative and quantitative research methods, economic analysis, and geospatial mapping). Another programme also found a significant self-reported improvement in knowledge of health topics discussed during the course (*N* = 28, *p* < 0.001) [39].

Four out of six included studies measured programme effectiveness in terms of improvements in sexual and reproductive health and rights research skills. Most scholars (82%, *N* = 18/22) involved in one online training programme reported having acquired new and practical skills [39]. Retrospective pre-test/post-test self-assessments from another online analytic skills course showed that combined skill levels improved by 1.75 out of 7 points on average (*N* = 23, *p* < 0.05) [37]. Another study reported that for the three modules, biomedical research, data collection in humans and analysis of human data, about 95.8% (*N* = 520/543), 94.0% (*N* = 604/643), and 96.3% (*N* = 419/435) of participants perceived the modules as having taught them a new skill and knowledge [41].

Authors reported specific improvements in scientific writing [36, 39], scientific methodology understanding [38, 40] plagiarism understanding, data analysis, and data interpretation [36, 39, 40]. Participants’ self-reported scientific writing abilities rose from 2.6 (SD =  ± 0.7) to 3.7 (SD =  ± 0.7) out of 5 points on average after taking part in a 6-week online mentorship programme (*N* = 28, *p* < 0.0001) [40]. Specific improvements in grammar, punctuation, and use of appropriate terminology were reported, as were improvements in participants’ abilities to search for and select appropriate articles and references for scientific writing in two studies (Total *N* = 51) [36, 39]. In terms of scientific methodology, pre-test/post-test assessments revealed improvements in participants’ understanding of the research process and of different study methodologies (i.e., observational studies, clinical trials, systematic reviews) (*N* = 28), as well as improvements in qualitative and quantitative data collection and analysis methods (*N* = 420) [36, 38, 40]. One six-week programme also measured and captured statistically significant improvements in self-reported soft skills including punctuality and attendance, initiative, attention to detail, critical thinking, and ability to self-organize and communicate effectively (*N* = 28) [39].

#### Improvements in self-efficacy and attitudes towards remote learning (level 3)

One study reported positive changes in participants’ self-efficacy and interest in distance learning over the study period. Pre-test/post-test self-efficacy scores, calculated based on participants’ responses to questions relating to their confidence to perform research-related tasks, improved by 0.8 out of 3 points on average across modules (*N* = 28, *p* < 0.0001) [39].

Most participants in one study [37] (78%, *N* = 18/23) reported that their attitudes towards distance learning for continuing education had improved because of the course, though no significant changes in participants’ beliefs regarding the usefulness of specific research skills were identified (*p* = 0.11). Of 22 scholars surveyed, 6 (27%) felt specifically that the programme motivated them to take a more research-oriented focus in their careers. Another study [41] reported that biomedical research module participants experienced a significant increase in self-efficacy 85.0% (*N* = 461/543). In addition, participants in data collection in humans and analysis of human data modules in the same study reported 75.5% (*N* = 485/643) and 81.0% (*N* = 352/436) improvement in self-efficacy.

#### Application of skills and research career advancement (level 4)

A majority of participants from four studies reported having applied the skills gained during online training during their own research activities after completion of the course [36–39]. Most participants of one online analytic skills course reported having shared knowledge/material from the course with co-workers informally (78%, *N* = 18/23) or via formal presentations (22%, *N* = 5/23) [39]. Comparing the number of times the same professionals had used specific skills in the six months before and after completing the course, all participants reported significant average increases in selecting appropriate secondary data sources (0.61, SD =  ± 0.84), conducting web searches (0.48, SD =  ± 0.85), and collecting (0.61, SD =  ± 1.03) and analysing (0.43, SD =  ± 0.79) qualitative data [41]. Of 174 surveyed participants of another online training course on sexual and reproductive health research, 67% (*N* = 118) had subsequently been involved in teaching, 74% (*N* = 129) had been involved in the design and/or implementation of a research project, and 98% (*N* = 171) reported having used the skills and knowledge gained from the course [40]. Additionally, another study reported that after completing each e-learning programme, 91.7% (*N* = 498/543), 89.2% (*N* = 573/643), and 94.0% (*N* = 409/435) of the three modules participants indicated an intention to modify the way they account for sex and gender in research [41].

Four of the included online training courses asked participants to report career advancements following participation [36–39]. For example, one study found that 46% (*N* = 81/174) of surveyed participants indicated they had received a promotion within two years of completing the course and 81% (*N* = 142) felt the course had contributed to the advancement of their career [37].

Two studies also highlighted the link between online training and subsequent opportunities for publication [36, 40]. In one study, 47% (*N* = 82/174) of scholars had published a scientific article within two years of course completion [40]; in another, 74% (*N* = 27/37) had published a manuscript or had a manuscript in peer review [36].

### Reported barriers and enablers to effective online training

#### Time management and workload

The most frequently cited barriers to effective online sexual and reproductive health and rights research training were related to personal organization and time management challenges, and participants’ competing professional obligations (Table 5). One study highlighted the difficulties in pacing activities and deadlines given the heterogeneity of participants’ external obligations [39]. As one example, the 15 h required to complete each of one programme’s, six data utilization modules was found to be incompatible with normal professional rhythms and may have contributed to high participant attrition [40]. Furthermore, authors emphasized the increased workload for lecturers who had to adapt to presenting remotely, as well as the added challenge for students to remain self-disciplined and self-driven with the remote format over the six-week long course.

**Table 5:** Summary of identified barriers and enablers to remote research training

Theme	Barriers	Contributing papers	Enablers	Contributing papers
**Time management and workload**	Scholars find online course load to be incompatible with competing professional commitments Organisers find the heterogeneity of external commitments makes it difficult to pace activities and deadlines Increased demand upon lecturers (to create remote education course content) and students (to remain self-disciplined and driven)	Farel 2001 [40]; Agarwal 2021 [39]	Course content that is clear and carefully planned, organized, and communicated allows participants to set appropriate goals and stay on top of activities and assessments Setting individualized targets and deadlines, and appropriate goals and breaking modules into smaller segments can facilitate a manageable workload and lower rates of attrition	Abawi 2016 [38]; Agarwal 2021 [39]; Farel 2001 [40];
**Mentorship and networking**	Organisers find the heterogeneity of time zones and countries limits real-time education opportunities	Abawi 2016 [38]	One-on-one mentorship, ongoing discussion and feedback, opportunities for informal and formal networking, and ability to engage in programme planning enables effective learning and application of skills Hands-on practice with feedback from faculty and staff provides helpful support and context for learning	Abawi 2016 [38]; Agarwal 2021 [39] Farel 2001 [40]; Santoro 2021 [37]
**Enabling environments**	Scholars find that lack of support and designated time to conduct research at home institutions, and exterior problems in work halt momentum to maintain and implement learned knowledge and skills over time	Brickley, et al. 2018 [36]	Selecting programme topics relevant to participants’ professional activities maximises successful implementation of new research skills	Abawi 2016 [38]

Despite these challenges, the flexible nature of remote training provided opportunities to overcome time and distance-related challenges. Cited ways to overcome scheduling challenges included providing participants clear indications of course content and expectations, learning objectives and organization [38, 39] setting realistic goals with mentors [40], breaking time-consuming modules into smaller segments, and generally ensuring a manageable workload [40].

#### Mentorship and networking

Three studies found that facilitating ongoing, one-on-one interactions between programme participants and mentors encouraged effective learning and application of skills [38–40]. Beyond curriculum-based interactions, student feedback from one study indicated that opportunities for mentorship through informal social interactions between facilitators and participants to the online course contributed to a sense of social inclusion, reduced power dynamics, and subsequently improved the learning process and outcome.

Opportunities to network with fellow scholars and establish future, potential collaborations [38] and to conduct hands-on application of research skills also enabled more effective learning [39]. Participants in both studies remained more engaged thanks to ongoing and regular feedback from programme faculty and mentors. Allowing participants to engage in programme design by selecting research topics relevant to their professional activities and practice also correlated with the rate of successful implementation of new research skills [37].

#### Enabling environments

One study ascribed identified barriers and enablers for the long-term use of the research knowledge and skills gained to the lack of an enabling research environment. For example, scientists who had participated in the International Traineeships in AIDS Prevention Studies (ITAPS) training programme found it difficult to publish research outputs due to competing time commitments and lack of support from their home institutions [36].

## Discussion

This systematic review summarizes the effectiveness, barriers, and enablers of remote education in SRHR research. Our search strategy identified six studies that met the inclusion criteria. Overall, participants enrolled in well-structured remote education programmes of a duration between 6 weeks to 12 months reported increases in knowledge and skills, as well as increased self-efficacy and attitudes towards this method of learning research skills. Enablers to remote education included flexibility offered by the format, as well as opportunities for mentorship and networking and application of skills. The participants taking part in the programmes expressed satisfaction with their experience, acquired new knowledge or skills, and remained engaged with the learning process as demonstrated by relatively high retention rates. This is in line with existing research on training of healthcare professionals remotely that indicates that structured remote education can achieve similar learning outcomes, satisfaction and engagement as traditional face-to-face learning environments [12, 42, 43].

As found in our review, the success of online learning programmes related to well-structured and build-for-purpose learning environments [44], coursework designed by experts in the field, and the promotion of social interactions [25, 45, 46]. A systematic review of the factors affecting e-learning in health sciences education found that e-learning may not be suitable for all disciplines or contexts [27]. In line with the published evidence on successful online education [27, 47], the identified enablers and barriers of effective remote SRHR research training relate to adequate planning, resource allocation, and social interactions. Previous research also showed that lack of planning in the design and implementation of online programmes can impede students’ ability to manage time and coursework [47]. Moreover, while the learning benefits of facilitating interactions amongst online scholars and facilitators during the course period have been widely cited [27], our results demonstrate an additional need for improved support from home institutions for applying skills after the completion of online programmes. And finally, our results also respond to existing theories in adult learning and online education, whereby autonomy, networking, and diversity are integral to success [16, 48, 49].

Encouragingly, our findings demonstrate that the remote format is considered by participants as both acceptable and effective for research methods training on SRHR despite the potentially sensitive nature of those topics. However, the studies included in this review mostly focused on specific research methods (both qualitative and quantitative) and few reported covering issues around ethics, gender, and rights relating to SRHR research. Further, given only two of the included studies disaggregated data on sex and gender, it is unclear whether our findings hold true for people of all genders. Gender and sex disaggregated data have been signalled as critical aspects to ensuring gender equality [50–53]. Given the known additional burden that women face outside the work environment, this may hinder their possibilities for remote education [54].

Also encouraging, internet connectivity and access to devices were not cited as a barrier to effective online SRHR research training among studies included in this review, as opposed to what others have found, specific to the COVID-19 pandemic [47]. However, the shift towards online, remote education, especially resulting from the COVID-19 pandemic forces us to address the issue of ensuring access to secure and fast connectivity across the globe, which is currently unequal [55]. None of the included studies used mobile devices and the evidence regarding efficacy of mobile devices for educational purposes is mixed and will be sensitive to timing of data collection in a fast developing area [19, 20].

Overall, the enablers and barriers identified to online learning in our review echo the relevance and applicability of recommended guidance for designing and monitoring SRHR research educational programmes [56] in the online context. Such a process requires defining the programme goal, describing optimal capacity to achieve the goal, determining existing capacity gaps, devising an action plan to fill the gaps and associated indicators of change, and adapting the plan and indicators as the programme matures [56].

### Strengths and limitations

To the best of our knowledge, this is the first review looking at the effectiveness of remote education in SRHR research. However, our review does have some limitations. First, the implications for policy resulting from this review are limited by the small number of studies meeting the inclusion criteria and the observational nature of the compiled evidence. Relatedly, we focused on peer-reviewed publications indexed in three large databases, but we did not explore the grey or white literature, where oftentimes reports on evaluations of training programmes are published. However, the small number of included studies in this review, did now allow for a robust meta-analysis and limits the generalisability of our findings. Nevertheless, the paucity of scientific publications on remote SRHR research training programmes is an important finding in itself, highlighting the need to make results of these efforts available to the scientific community through peer-reviewed articles. Second, half of the included studies focused on participants from high-income countries. However, participants from the remaining studies were from a wider variety of countries. Additionally, two of the six included studies were of ‘blended-learning’ format, and it was not possible to differentiate positive outcomes relating to the online versus face-to-face components. Finally, we did not find any evidence of shorter online courses or from MOOCs, meaning we cannot assure our findings can also be extended to those types of courses.

## Conclusion

The available evidence is limited but demonstrates the suitability of the remote learning format for providing courses on SRHR research methods and content, when course learning objectives and expectations are outlined clearly, and ongoing mentorship provided. Online learning opportunities may also help overcome financial and geographic barriers in accessing training on SRHR research. There remains a need to document results of online learning, in particular in LMICs, to further provide proof that remote education in SRHR research can sustainably replace in-person training.

### Supplementary Information

**Additional file 1.** PRISMA 2020 Checklist.

**Additional file 2.** Quality Assessment Checklist for Before & After Studies.

## Data Availability

The datasets used and/or analysed during the current study are available from the corresponding author on reasonable request.

## Abbreviations

RCS: Research Capacity Strengthening
SRHR: Sexual and Reproductive Health and Rights
SAGER: Sex and Gender Equity in Research
MOOC: Massive Open Online Course
ITAPS: International Traineeships in AIDS Prevention Studies
LMIC: Low- and Middle-Income Country
HRP: Human Reproduction Programme
WHO: World Health Organization
UNDP: United Nations Development Programme
UNICEF: United Nations International Children’s Emergency Fund
UNFPA: United Nations Population Fund
PRISMA: Preferred Reporting Items for Systematic Reviews and Meta-Analyses
MCH: Maternal and Child Health
